# Virtual Reality Pornography: a Review of Health-Related Opportunities and Challenges

**DOI:** 10.1007/s11930-022-00352-9

**Published:** 2022-11-21

**Authors:** Leighton Evans

**Affiliations:** Department of Media and Communication, Digital Technium Building, Swansea University, Swansea, SA2 8PP Wales UK

**Keywords:** Virtual Reality, Pornography, Arousal, Empathy, Teledildonics

## Abstract

**Purpose of Review:**

Virtual reality (VR) pornography is a relatively new medium for the experience of pornography. In juxtaposition with traditional modes of experiencing pornography, such as two-dimensional (2D) displays, VR promises a new experience of pornography for the user. VR can offer the feeling of ‘being there’: an increased sense of immersion and presence in a mediated experience thanks to the sensory affordances of the medium. In an effective VR environment, the user is immersed in the experience itself, feeling an embodied presence in the world presented to them and able to interact with the environment and others in the environment in ways that cannot be achieved in other media. In terms of pornography, this is potentially revolutionary. The user can be embodied in one of the performers and experience a unique perspective. Alternatively, there are interfaces that will allow for the performer and viewer to physically interact with one another and experience physical arousal from the actions of the other at a distance. The possibilities of VR pornography are therefore related to the intensity of experience, the changing relationship with the performers and others in pornographic media, and the possibility of new, embodied experiences of arousal utilising networked, embodied technologies. This research review assesses to what extent research on VR pornography has supported these possibilities, affordances, and developments.

**Recent Findings:**

23 articles were included in the present review. Findings demonstrate some increases in arousal and empathy in using VR pornography. However, further empirical evidence for these findings is still needed. In addition, teledildonic technology is lacking empirical research and the effects of the use of this technology in conjunction with VR requires research.

**Summary:**

Collectively, the results underscore the notion that VR improves immersion and presence for subjects, and this can translate to increased sexual desire, empathy for performers in pornography, and sexual anxiety in watching pornography. This is a field in infancy, and the initial results of empirical work suggest that VR can intensify some key aspects of the experience of pornography. Theoretical reflections on VR pornography indicate many areas that require further empirical research.

## Introduction

Virtual reality (VR) has been proposed as a potentially revolutionary medium for pornography [1]. In popular discourse, VR pornography has been identified as being a development which may reshape the relationship between performer and viewer in pornography, and as a mode of experiencing pornography which will add intensity and a feeling of presence to the viewing of pornographic material [2]. Additionally, it is argued that the increased realism of VR pornography will revolutionise the experience of pornography and will be a significant driver of early VR business [3, 4]. Pornography makes up more than 20% of all Google searches, and VR videos are watched more frequently in comparison to regular videos when corrected for the number of videos uploaded [5]. As befits a relatively new technology, the range of content in VR pornography is more limited than in 2D pornography and the creation of amateur/non-mainstream VR pornography is limited by the costs and use of specialist cameras for the production of VR pornography [1]. The change of focus for performers and producers in VR pornography compared to some other traditional forms—from the co-star to a tight focus on the camera to focus on the viewer in VR—illustrates a critical aspect of the difference between VR pornography and 2D pornography. While point-of-view (POV) pornography has existed for many years, the use of a VR headset facilitates a new positionality for both viewer and performer of pornography based on greater control of the field of vision, and the interfacing of VR headsets and complimentary technologies that simulate touch and co-presence with the performer offers a new paradigm of mediated sexual behaviour [1]. The proposition underpinning the potential for VR pornography is that the technology can be more arousing and intimate than traditional 2D pornography, which has already been found to have a considerable effect on arousal [6–11]. Beyond the immersion created by a VR headset, there are several different types of teledildonic technologies which can sync with VR experiences. Companies such as Lovense and Kiiro now offer a range of teledildonic technologies for this purpose. These include fleshlights for men, vibrators, prostate massagers, and anal plugs [12–14]. The syncing of the VR experience with the stimulation received from these devices can allow for a physiological effect on both performer and audience member in synchronous real time. During the COVID-19 pandemic and subsequent social distancing, there was a significant increase in the sales of these devices [15, 16]. However, some research has argued that teledildonic sexual activity may be considered a mediation of sexual behaviour that veers too close to infidelity [17]. The relative sensory focus and affordances of presence of VR may have therefore a significant effect on arousal and empathy as a factor in the performer-audience relationship. The notion of VR as an ‘empathy machine’ has led to predictions that VR will facilitate more empathetic relationships between pornography and the viewer and pornographic actors and viewers. This affective turn in VR pornography is contingent upon the use of teledildonic technology to facilitate new embodied relations for the pornography viewer and performer.

## Method

A research review was conducted of recent papers of a quantitative, qualitative, and theoretical nature on VR pornography published between January 1, 2017, and September 1, 2022. The research method derived from and followed methodologies of systemic and organised research reviews [18–21]. Pornography and VR search terms (‘VR pornography’, ‘virtual reality pornography’, ‘cybersex’, ‘porn’, and ‘teledildonics’) were searched to identify relevant works in Google Scholar, Scopus, Web of Science, and PubMed. A review of references in related works also identified some relevant studies. This review included quantitative empirical studies, qualitative studies without quantitative or empirical components, and theoretical or systematic reviews to address the lack of empirical work identified over the period. Works which specifically identified and focused upon the use of and effects of VR pornography were included in the review. Works which only tangentially mentioned VR pornography in relation to cybersex or other phenomena were not included in the reviewed research unless the results contributed to the discussion on arousal or empathy. These studies provide a context for current research on VR pornography and suggestions for further research. This search is an initial indicator that, while some empirical work exists in the area, there is a wide epistemological gap thanks to the lack of empirical work in the area.

## Results

After screening out studies that did not meet the inclusion criteria, a total of 23 empirical, quantitative studies and non-empirical studies published between January 1, 2017, and July 1, 2022, were identified that were relevant to the present review. Other research in this review is used to contextualise the findings and insights into VR pornography in the wider research context around pornography and cybersex (Table 1).

**Table 1:** Summary of all studies included in the present review

Study	Type of research	Characteristics	Key outcomes	Summary of findings
Arrell [46]	Review	Chapter that surveys some of the philosophical issues raised by the increasing integration of physical and Internet-mediated technologies into our sex lives	Teledildonics can help overcome spatial separation in intimate relationships	Some emergent (bio)technologies can help overcome obstacles that can get in the way of a happy sex life
Asci et al. [5]	Review	Review of consumption of VR pornography	VR pornography is watched more frequently compared to regular videos when the sample is corrected for number of videos uploaded	VR pornography is new in the porn industry, and the number of videos uploaded is low. VR porn seems to be attracting more watchers; this trend might continue in the future
Ashton et al. [29]	Review	Assessment of how new digital technologies will change definitions and practices of researching pornography	VR pornography can alter and improve the experience of pornography for the user	Thanks to changes in realism, immersion and interactivity VR pornography may be an improved experience for users
Bollmer [32]	Non-empirical	Critical analysis of the possibility of empathy from VR	VR is not capable of engendering real empathy	Any technologies intended to foster empathy merely presume to acknowledge the experience of another but fail to do so in any meaningful way
Dekker et al. [23••]	Empirical quantitative	50 male participants in a repeated measures design, recruited via a local website in Germany	VR pornography induced feelings of intimacy with performers	In the VR condition, participants felt more desired, more flirted with, and more looked into the eyes. They were also more likely to feel connected with the actors and more likely to feel the urge to interact with them
Elsey et al. [25••]	Empirical quantitative	Cross-sectional study of 95 heterosexual participants (47 female, 48 male) in a repeated measures design recruited via university online system and snowball sampling in the Netherlands	Men found VR pornography more arousing than 2D scenes, but this was not the case for women	VR consistently elicited a greater sense of presence than typical pornography, and presence was positively correlated with sexual arousal
Evans [1]	Review	Critical review of VR pornography and teledildonics	Teledildonic technology is heteronormative and male-focused	VR pornography and teledildonic technology have significant issues with inclusion
Faustino [42]	Non-empirical	Critical reflection on teledildonics from a materialist perspective	This technology reinforces the ‘coital imperative’, by equating sexual interaction with penetration of the vagina by the penis	Although teledildonics may permit other formulations, specifically for non-heterosexual couples, the penetrative act remains a presupposition of this technology
Flore and Pienaar [40]	Non-empirical	Case studies of sex toys to examine how the relationship between data and sexual subjectivity is being transformed through these emerging technologies	Sexual practices, intimacy, and pleasure become ‘datafied’ through these sensory technologies	The datafication of sexual practice has significant impacts on privacy and the ownership of sexual pleasure
French and Hamilton [31]	Empirical quantitative	195 men (mean age 19.84, s.d. 2.7) and 310 women (mean age 19.8, s.d. 3.8) from Eastern Canada completed an online questionnaire. Participants were recruited through Facebook, Kijiji, and community advertisements. 63% of the samples were introductory psychology students	Women show a preference for female-centric pornography consumption (typically that which depicts more genuine female pleasure, natural bodies, attractive male leads, and greater context)	Although effect sizes were small, women who reported viewing pornography with more female-centric features also reported more positive effects of pornography on sex life and perceptions of the other gender
Gesselman et al. [45]	Empirical quantitative	A web-based, demographically representative sample included 7,512 American adults aged 18–65 years, with a near-even gender split of men/women and moderate racial diversity (63% White)	Participants who were younger, were men, had higher income, and were sexual minorities reported more frequent engagement with all forms of sextech assessed	Participants indicated their engagement with eight forms of sextech, including teledildonic use and accessing virtual reality pornography as well as two more common domains (online pornography and sexting). Engagement with pornography and sexting was high, but some demographics indicated increasing engagement with new sextech
Kaisar [41]	Non-empirical	Critical examination of how the relationship between data and sexual subjectivity is being transformed through these emerging technologies	Teledildonics are marketed as a substitute for heteronormative sexual encounters whereas they are closer to a simulated experience of mutual masturbation	Intimate encounters between interactive sex toys and bodies should be considered complex technological and biological assemblages, where machines and the human body come into intimate connection through datafication
Lafortune et al. [26••]	Empirical quantitative	39 participants allocated into low (*n* = 16) or high sexual aversion (SA) (*n* = 23) groups. Mean age was 29.9 years (s.d*.* = 11.31). The sample was mostly comprised of women (> 60%), with 17% of participants identifying as men and 21.5% as non-binary. Participants were recruited from a previous SA study and through social media in France	High-SA participants showed increased discomfort as a factor of time exposed to VR pornographic performance by a synthetic actor	Subjective measures of discomfort were increased significantly in high-SA participants through increased exposure to a synthetic character displaying erotic behaviours in a virtual room
Liberati [38]	Non-empirical	Phenomenological assessment of teledildonic possibilities	Teledildonics have the potential to re-shape our living body and, in so doing, re-shape our affections as well as our perception of the world	Teledildonics provide tactual sensations that simulate part of a subject’s body as being relocated in another place, enabling a subject to ‘connect’ and to ‘play’ with a second subject as if they were actually in the same place at the same time, in other words, to engage in remote sexual activity
Liberati [39]	Non-empirical	Critical analysis of the effects of the introduction of teledildonics on sexual lives according to postphenomenology and mediation theory	Teledildonic use will have a transformative effect on the scope and range of human sexual relations and human-object sexual relations	Teledildonics will allow human beings to have sexual intercourse with every object around by turning them into sexually interactive ‘quasi-others’. This will affect the way we give meanings and values to love and sex in general
Marcotte et al. [37]	Empirical quantitative	Survey of 8004 American adults, mean age 44.05, 47.8% male, 51.2% female, 1% other	People with mental health struggles may be drawn to interactive, digital forms of sexual behaviour as a means of alleviating symptoms through distraction or self-soothing	People with higher anxiety and depression were more likely to engage in sextech. However, those who were lonelier were less likely to engage with sextech, suggesting the aforementioned patterns were not due to lack of social connection
McArthur and Twist [47]	Review	Review article of issues associated with emerging sexual technologies	A framework for understanding the nature of digisexuality and how to approach it is imperative for clinicians and researchers	Many practitioners are unfamiliar with new technologies like teledildonics, as well as the social, legal, and ethical implications of this technology
Milani et al. [24•]	Empirical quantitative	38 female subjects in a repeated measures design, recruited via university mailing list in Canada	VR can induce feelings of sexual presence and presence more generally	With medium to large effects, general presence, sexual presence, and sexual arousal were significantly higher for VR videos relative to 2D videos
Orel [44]	Review	Critical review and exploration of the potential of VR pornography	VR pornography has the potential to reshape sexual desire	The affordances of VR pornography allow creators to create a new reality, which will allow for new expressions of desire and sexual activity
Rubin [27]	Review	Review of VR as a technological medium	VR pornography may increase empathy with performers and transform viewer-performer relationship	Capacity of VR for immersion and presence will transform the relationship between the VR consumer and performer into an empathetic, intimate one
Simon and Greitemeyer [22•]	Empirical quantitative	60 male participants in a repeated measures design in Austria, recruited via university mailing list	VR technology was found as consistently more arousing when displaying pornography than 2D displays	Results showed that viewing pornographic video material via VR technology had a stronger effect on psychophysiological reactions as well as subjective experience than using the conventional desktop display
Sparrow and Karas [43]	Non-empirical	Examination of the legal ramifications of teledildonic technologies	There are risks with using Internet-enabled prosthetics around consent and the identity of one’s partner	If one is unsure about who one is having virtual sex with then it is possible that the user would become the victim of rape by deception. This raises difficult questions about the definition and significance of sexual intercourse and virtual sex
Wood et al. [30]	Empirical qualitative	24 male, 18 female, and 3 other gendered participants recruited via fan fiction forums, Reddit forums, and on social media	Participants produced stories of the ‘perfect’ pornographic experience after using VR porn and reproduced heteronormative and hegemonic masculine tropes	The common cultural ideal non-experts constructed of a ‘new’ pornographic experience through use of the ‘Story Completion Method’. The stories reproduced ideals around heteronormativity and hegemonic masculinity

### Demographics and Methodologies of Studies

Of the 9 empirical studies, research was distributed across countries. Two studies were conducted in Canada; one in Austria, France, the Netherlands, and Germany; two large-scale general sexual behaviour survey in the USA; and one study using a multi-national sample utilising social media.

All empirical studies used adult samples. Two studies used samples limited to men [22•, 23••]. One study had an exclusively female sample [24•]. The remainder of the empirical studies had male, female, and other gendered participants. There was a preponderance of studies identified using limited sampling methods (e.g. cross-sectional designs that relied exclusively on snowballing samples or undergraduate samples), and longitudinal designs were not identified.

Non-empirical studies identified as being relevant to the aims of this review utilised a range of critical theoretical positions, including phenomenology, postphenomenology, materialism, critical feminist, and critical data study approaches.

### Measurement

Empirical studies used a combination of subjective self-reporting and physiological data to assess arousal, intimacy, empathy, and anxiety when assessing the differences between VR pornography and traditional 2D pornography. Non-empirical research based on critical theory used a range of critical perspectives on the development, use, and effects of VR pornography.

### Arousal

Research on arousal has assessed whether VR pornography invokes more physiological arousal than traditional 2D pornography [6–11]. This research has two important implications. Firstly, from a theoretical perspective, an increase in immersion and presence aligned to an increase in arousal speaks to the efficacy of pornography in VR. Secondly, arousal in VR is important in terms of therapeutic interventions for sexual disorders and anxieties. Individuals with sexual aversion anxiety disorders, for instance, could be assisted by VR therapy if VR stimulates arousal and can be used in a therapeutic capacity to safely reduce anxiety through immersion therapy. Conversely, if VR pornography is more arousing than 2D pornography, this could have significant implications for addiction to pornography and other problematic behaviour associated with pornography consumption.

From the limited empirical research conducted, there is evidence that VR is more arousing than other media for experiencing pornography. Three research studies support this claim. Research has supported the notion that arousal increases with VR stimuli with an exclusively male sample [22•]. Sixty male participants were alternately shown sexually explicit video material on a two-dimensional desktop monitor and a three-dimensional, high-immersive VR head-mounted display (HMD). Physical arousal was continuously measured as skin conductance response, whereas subjective sexual arousal was measured using a slider. Questionnaire measures of subjective sexual arousal, presence, and sexual presence were also employed. With medium and large effect sizes (*ƞ*^2^ = 0.12–0.47), the results indicated a stronger effect on physiological and subjective sexual arousal when men viewed videos using VR compared to when videos were viewed using the 2D desktop display, and presence and sexual presence were also fostered in the higher immersive VR condition. There was a positive correlation noted between presence and subjective sexual arousal in the VR condition but not in the desktop condition. For sexual presence and subjective sexual arousal, a positive correlation was found for both conditions. In analysis, controls for order of exposure were used and no significant impact of order on the dependent variables was reported except for physiological arousal. Higher levels of physiological arousal were seen during the second exposure regardless of stimulus modality. The results showed that viewing pornographic video material via VR technology had a stronger effect on psychophysiological reactions as well as subjective experience than using the conventional desktop display. As women were not included in this study, it leaves open the question as to how VR immersion impacts women’s sexual response. Additionally, the study used a student population which raises questions with regard to the applicability of the research to older populations.

These observations have been partially confirmed in research which has addressed female arousal as well as male arousal [25••]. Ninety-five heterosexual participants recruited via university online system and snowball sampling in the Netherlands (48 males and 47 females) viewed either VR or 2D pornography (between subjects), from both voyeuristic and first-person perspectives (within subjects), and rated their subjective arousal and presence. Men found VR pornography more arousing than 2D scenes, but this was not the case for women. This effect was small (*ƞ*^*2*^ = 0.04), indicating a gender and modality (VR vs. 2D) interaction, as men showed higher subjective sexual arousal to VR relative to 2D, but women did not. Rather than VR being generally experienced as more arousing, a first person or ‘participant’ perspective consistently induced greater arousal relative to a voyeuristic view, regardless of presentation medium in both genders. VR did elicit a greater sense of presence than typical pornography, and presence was positively correlated with sexual arousal. The findings indicate that VR can enhance the experience of arousal and pleasure in response to pornography, but this is gender-contingent in terms of effect.

Research on arousal specifically focusing on women has also been conducted [24•]. The research used high quality, women-centred erotica and examined whether stimulus modality (VR vs. 2D) and point of view (POV: first-person vs. third person) impacted women’s feelings of sexual presence (activation of sexual response induced by the perception of being present), sexual arousal, and sexual desire (dyadic and solitary). Results from 38 women indicated that with medium to large effects, general presence, sexual presence, and sexual arousal were significantly higher for VR videos relative to 2D videos. Sexual presence was higher for first-person POV than third person POV. These results parallel the findings of research specific to men and indicate that VR pornography experiences elicit a sense of ‘being there’ and a consequential increase in sexual arousal. As this research relied on correlational data, there is an issue with causality. While the greater immersion into the VR films triggered increases in sexual arousal as well as sexual desire, there is a possibility that erotica-induced increases in sexual arousal and desire triggered the increase in sexual presence. Again, the study used a student population which raises questions with regard to the applicability of the research to older populations.

Research has also considered the increased arousal potential of VR pornography in people with sexual aversion (SA) anxiety [26••]. This study focused on sexual aversion (SA) defined as the experience of fear, disgust, and avoidance when exposed to sexual contexts or cues. The study was aimed at validating a virtual environment’s ability to progressively trigger the typical emotional responses of SA. The sample was mostly comprised of women (> 60%), with 17% of participants identifying as men and 21.5% as non-binary. The thirty-nine participants (16 low-SA and 23 high-SA individuals) were immersed in a virtual room using a VR headset and then successively exposed to six scenarios in which a synthetic character showed erotic behaviours of increasing sexual intensity. Throughout immersion, subjective measures of anxiety and disgust, skin conductance, heart rate, cardiac output, and eye movements increased. The changes in SUDS and physiological variables were examined through repeated measures analyses of variance. SUDS scores significantly increased as the levels of exposure progressed among the high-SA participants, who also reported significantly more anxiety and disgust than the low-SA group. This research indicates that VR may be a promising tool for as part of a virtual reality exposure-based treatment for SA and this should be tested further.

### Empathy

An argument has been developed in non-empirical literature that proposes that VR pornography may be a more humanising and less objectifying form of pornography than conventional pornography [27]. This argument is contingent on the idea that using VR can provide an empathetic aspect of ‘being there’ or being embodied (on the part of the user) in the scene that could reduce the voyeuristic aspect of pornography. Empathy through sincerity and directness has been effective in creating enduring audiences in 2D pornography [28]. Voyeurism could be replaced with a participatory intimacy that will increase empathy and decrease desensitization and detachment with regard to the subject position of the viewer in comparison to the performer. There are several ways that VR could alter and even improve the experience of pornography: veridically (the experience is life-like to the user), through immersion (the consumer becomes integral to the pornography and feels part of the experience), interactively (the consumer’s decisions and actions determine what occurs in the scene), and through unboundedness (any pornography is available, no matter how particular, bizarre, or extreme) [29]. While the visual representation of VR pornography experiences could reasonably be described as ‘pornographic’, when technology facilitates interactions between two people using virtual space, it is not pornography but is better described as a sexual encounter or, if money is exchanged, sex work [29].

One empirical study has tested these assumptions [23••]. Fifty male participants watched two pornographic films on consecutive days in a laboratory setting, one in VR and one traditional 2D film. The perception of the films was assessed using self-report measures and through the measuring of oxytocin (a social neuropeptide associated with facilitating intimacy and interaction) production. In the VR condition, participants were more likely to feel connected with the actors and more likely to feel the urge to interact with them. Participants were also more likely to rate the IQ of actors in VR as higher than in 2D films. Saliva levels of oxytocin were related to the perceived eye-contact with the virtual persons indicating a role for the social neuropeptide in the perception of increased intimacy and interaction in VR. In this research, VR pornography appears to elicit the illusion of intimate sexual experiences. Whether this can be considered as commensurate with empathy is open to question, although there is clearly a deeper relationship with the performer.

This study only has male participants, so generalising from the research is questionable. However, this resonates with non-empirical observations on empathy in VR pornography. Research utilising materialist critical theory has claimed that VR pornography forces the viewer into the subject position of a straight, white male [1, 27] and the experience of VR pornography is of an (often) white female submitting to the sexual desires of the male actor. The result of this is that VR pornography reproduces ideals around heteronormativity and hegemonic masculinity in pornography, which would affect the possibility and nature of empathy [30]. Empirical research has found that female-centric pornography consumption (typically that which depicts more genuine female pleasure, natural bodies, attractive male leads, and greater context) was particularly associated with more positive effects on women’s sex lives [31]. Masculine pleasure-focused content is unlikely to address these effects. Other researchers have argued that any technologies intended to foster empathy merely presume to acknowledge the experience of another but fail to do so in any meaningful way [32].

### Teledildonics

A further affordance of VR technology not covered in previous research is the use of teledildonics. Teledildonics allow for a sexual experience with other people at a distance by creating an interface between the two (or more) people that stimulates sexual pleasure either synchronously or for one party [33]. The concept of teledildonics first emerged in the 1990s but has become more prevalent with the emergence of VR as a consumer medium [34, 35]. Research has indicated that technologically mediated sexual interactions have the potential to improve sexual wellbeing in some populations [33, 36] Teledildonic devices, such as remotely controlled dildos, provide tactile sensations that simulate part of a subject’s body on the behest of another person while connected digitally, enabling a subject to interact with a second subject as if they were in the same place at the same time. This can create a feeling of ‘being there’ through sensory biofeedback as well and auditory and visual immersion [1, 35]. Empirical research has found that people with mental health struggles may be drawn to interactive, digital forms of sexual behaviour as a means of alleviating symptoms through distraction or self-soothing [37]. From a phenomenological perspective [38], the person using teledildonic technology can freely define gender, use the teledildonics to record their actions, and change them by translating them as they please. In effect, teledildonics can allow human beings to have sexual intercourse with any appropriately networked object around by turning them into sexually interactive ‘quasi-others’ [39], and this change will affect the way we give meanings and values to love and sex in general.

The use of teledildonics and the residual data that these devices will produce has been identified as a major potential issue from critical feminist perspectives [40, 41]. Sexual practices, intimacy, and pleasure become ‘datafied’ through these sensory technologies, and this data production may have profound implications for privacy. Another major issue identified with teledildonics from a materialist position is that this technology reinforces the ‘coital imperative’, by equating sexual interaction with penetration of the vagina by the penis [42]. Although teledildonics may permit other formulations, specifically for non-heterosexual couples, the penetrative act remains a presupposition of this technology. The prioritisation of male desire does not address the spectrum of embodied sexual experiences that are possible. The use of teledildonics for intimacy is also fraught with issues. There are risks with using Internet-enabled prosthetics around consent and the identity of one’s partner [43]. If one is unsure about who one is having virtual sex with, then it is possible that the user would become the victim of rape by deception. This raises difficult questions about the safety of sexual intercourse and virtual sex. Moreover, one can already envision a multi-tiered hierarchy of intimacy, where wealthy users can afford sophisticated haptic systems designed for physical intimacy while basic users have little but their words and the non-verbal communications of their avatar to express their emotions. One should not presuppose that teledildonic technology will even be normally distributed, let alone ubiquitous.

## Discussion

VR pornography appears, on the basis of limited research, to increase sexual arousal with regard to the reaction of people to pornography. Studies that have compared 2D pornography to VR pornography have identified increased subjective and objective levels of arousal in participants for VR pornography. Moreover, this increased arousal is also seen in sexually adverse participants, with VR pornography increasing levels of reported anxiety and physiological arousal compared to 2D pornography. On the basis of one study, empathy with pornography performers on the basis of increased feelings of intimacy is higher with VR pornography. With regard to teledildonic technology, there is only theoretical discussion of the possible effects of this technology on sexual desire, behaviour, and arousal.

## Conclusions

The implications of a limited set of empirical studies are difficult to summarise without caution. However, this early research does indicate that VR pornography has both the potential and the current effect of being a more stimulating form of pornography consumption, both physiologically and psychologically, and may play a role in reshaping sexuality, the expression of sexual behaviour and desire [44]. There are several potential implications of this set of findings. The use of VR pornography could have effects on addiction to pornography and associated behaviours thanks to increased stimulation. VR pornography could also play an important role in interventions around pornography addiction thanks to the potential humanising effect of the increased presence and immersion felt by users. VR pornography may have a role to play in immersion therapy-based interventions around sexual anxiety and other related social anxiety disorders. This research on the efficacy of VR pornography compared to 2D pornography does not consider the effects on performers (particularly in synchronous pornography where teledildonic technology can be used to interact with performers), production, the content of VR pornography, or use patterns of VR pornography.

All these areas are relevant to future research agendas on VR pornography as they indicate considerable gaps in the current literature. While research on VR pornography is in an early stage, so is the use of VR for pornography. Considerable issues have been identified with regard to the heteronormative and male-focused nature of both VR pornography and teledildonics, and research does indicate that younger, higher income men report far more use of teledildonics and other emerging forms of sexual technology [45]. Further research is needed to understand what the specific demographics of use are for teledildonics synced with VR, particularly if the potential for teledildonics to overcome spatial separation between people is to be harnessed [46]. If these are continuing issues, then the use of VR pornography in research and treatment may be limited, as may the general use of VR pornography. Additionally, there are major lacunas in the research regarding the use of VR pornography by minors and adolescents. All these areas require further empirical research if clinicians and researchers are to be prepared for the challenges and benefits associated with the adoption of such sexual technologies [47].
